# Prevalence of Depression Among Undergraduate Medical Students in India: A Systemic Review and Meta-Analysis

**DOI:** 10.7759/cureus.33590

**Published:** 2023-01-10

**Authors:** Gitashree Dutta, Navin Rajendran, Tarun Kumar, Shoban B Varthya, Vinoth Rajendran

**Affiliations:** 1 Community Medicine and Family Medicine, All India Institute of Medical Sciences, Jodhpur, IND; 2 Paediatrics, Dr. Sampurnanand (SN) Medical College, Jodhpur, IND; 3 Pharmacology, All India Institute of Medical Sciences, Jodhpur, IND

**Keywords:** india, systematic review and meta-analysis, medical students, depression, prevalence study

## Abstract

Background: Systematic reviews have proved that there is a high rate of depression among medical students when compared with their age-matched peers. Very few studies have evaluated the pooled prevalence of depression among medical students in India.

Objectives: To determine the pooled prevalence of depression among medical students in India.

Materials and methods: This review was done by searching databases like PubMed, Google Scholar, and Scopus for available original articles published between 2019 and 2022 on depression among Indian medical (MBBS) undergraduate students using PRISMA guidelines.

Results: A total of 19 original research articles were included in this review, involving students at different medical colleges from various regions of India. The pooled prevalence of depression among 5944 medical students was 50.0% (95% CI: (31%-70%)) based on the random effect model. This meta-analysis also found that the pooled prevalence of depression among females (pooled prevalence: 38.0%, 95% CI: 20.0 to 58.0) was slightly higher than among males (pooled prevalence: 34.0%, 95% CI: 15.0 to 55.0).

Conclusion: The high prevalence of depression among medical students demands regular screening for depression along with counselling services. It shows that there is a need to raise awareness among students and other stakeholders, such as parents and medical educators, concerning symptoms and signs of depression among medical students.

## Introduction and background

The World Health Organization (WHO) defined health as "a state of complete physical, mental and social well-being and not merely the absence of disease or infirmity", a definition relevant today [1]. Further, it is stated that "not depressed" is not the end goal as there is a spectrum of well-being, with the disease at one end and optimal well-being at the other. The World Health Organization recognizes mental health disorders as important causes of morbidity and disability, with depression as one of the leading causes of mental health disorders.

In 2016, the age-standardized prevalence of depressive disorders in South Asia was 3.9% (95% UI: 3.6 - 4.2%), 3.7% (95% uncertainty interval (UI): 3.4-4.1%) in Bhutan, 3.9% (95% UI: 3.6-4.2%) in India, 4.4% (95% UI: 4.4-4.8%) in Bangladesh, 3.0% (95% UI: 2.8-3.3%) in Pakistan, and 4.0% (95% UI: 3.7-4.3%) in Nepal [2]. Systematic reviews have proved that there is a high rate of depression or depressive symptoms (27.2%) among medical students when compared with their age-matched peers before the COVID-19 pandemic [3,4]. All people, including medical students, would benefit from being aware of where they are on the well-being spectrum and what they can do to climb up the spectrum. Students who experience depression also experience additional mental health issues like anxiety, burnout, substance abuse, and suicidal thoughts. Undergraduate students' mental health is a significant public health issue on a global scale [5-8].

The COVID-19 pandemic has taken a toll on the mental health of different individuals worldwide for various containment measures and the disease itself [9]. During the COVID-19 pandemic, college students are more prone to mental health disorders along with COVID-19 patients and medical personnel. Compared to other training programmes, medical education has the highest academic and emotional requirements. A systematic review and meta-analysis with research works published globally show depression prevalence of 37.9% among medical students [10].

The pooled prevalence of depression based on standard screening instruments among medical students in India was 40% before the COVID pandemic [11]. However, no studies have been conducted to date evaluating the effect of this healthcare crisis on medical trainees in India during the pandemic. This meta-analysis includes cross-sectional studies on depression among medical students in India to determine the pooled prevalence of depression among medical students in India.

## Review

Materials and methods

This study was completed following the PRISMA checklist [12], and it was registered in PROSPERO (CRD42022331012) [13]. Before beginning the literature search, the study's framework was created using PRISMA criteria; after that, no changes were made.

Literature search

A systematic search was undertaken in three databases, namely PubMed, Google Scholar and Scopus, for all the available articles published in the English language during 2019-2021 on the prevalence of depression amongst undergraduate medical students in India by two independent investigators. (Appendices 1) Based on the "PICOS" principle for formulating the search strategy, the search terms include "Depression", "Undergraduate students", "medical students", "undergraduate medical students", and "MBBS students". We have added relevant articles identified by several databases to the search using Rayyan software. It was also attempted to contact the authors of articles whose full texts were unavailable in the databases. Appendices 1 presents a comprehensive search strategy.

Inclusion and exclusion criteria

The inclusion criteria were 1) Cross-sectional study design, 2) The study population was undergraduate medical students from India, 3) Assessing the prevalence of depression using a standardized instrument, and 4) The study period was from January 2019 to April 2022. The articles which did not state the information on the prevalence of depression or outcome data among the undergraduate medical students were excluded.

Study selection and data extraction

The studies were retrieved based on the search strategy discussed a priori. Multiple publications of the same study were identified and collated. Two independent reviewers screened titles and abstracts to identify the studies that meet the inclusion and exclusion criteria. Any disagreements were resolved by discussion or by a third reviewer. Data were extracted using a pre-designed spreadsheet from the studies which included the first author name, year of publication, study period, study setting, sample size, the instrument used for assessing depression, the total number of medical students, number of females, number of males, age (mean) of the student, prevalence of depression overall, and among male students and female students.

Quality assessment/risk of bias (ROB)

Using the Joanna Briggs Institute's (JBI) Critical Appraisal tool for systematic reviews of prevalence studies, we evaluated the study's methodology which was shown in Table 1 [14,15]. The reviewers independently assessed the ROB using the Modified JBI criteria. In case of a mismatch of results, the discrepancies or disputes have been checked, and the reviewers came to a common opinion by discussing it with a third investigator. The evaluated articles were divided into three categories: high ROB (JBI score 49%), moderate ROB (JBI score between 50% and 69%), and low ROB (JBI score >70%) [16].

Data synthesis and analysis

In this meta-analysis, to determine the prevalence of depression among medical students, the pooled estimates with 95% confidence intervals (95% CI) were calculated using the random-effects model (DerSimonian-Laird method) due to high heterogeneity [17]. The forest plots have been used to determine the prevalence of pooled estimates. The estimation was calculated using MetaXL version 5.3 and an Excel spreadsheet. A P-value less than 0.05 was considered significant. The Q and I^2^ statistics were used as tests for heterogeneity. Q test with P < 0.10 was considered statistically significant heterogeneity and I^2^>75% was labelled as high heterogeneity [18]. This review used the Doi plot and the LFK index for publication bias. The double arcsine prevalence was taken as the x-axis for the funnel plot. Arcsine transformation was required with data with extreme values like 0 or 1. Otherwise, the confidence interval for the proportion could include values outside the range of 0 and 1, representing 0% and 100%. The y-axis of the graphic was set to the precision obtained from the inverse of Standard Error (SE). Doi plot and the LFK index for publication bias were used to validate the funnel plot's asymmetries. Values describe the publication bias over ±1 of the LFK index [19]. Sensitivity analysis was done to indicate the major determinant for the pooled prevalence of depression.

Results

Study Characteristics

**Figure 1:**
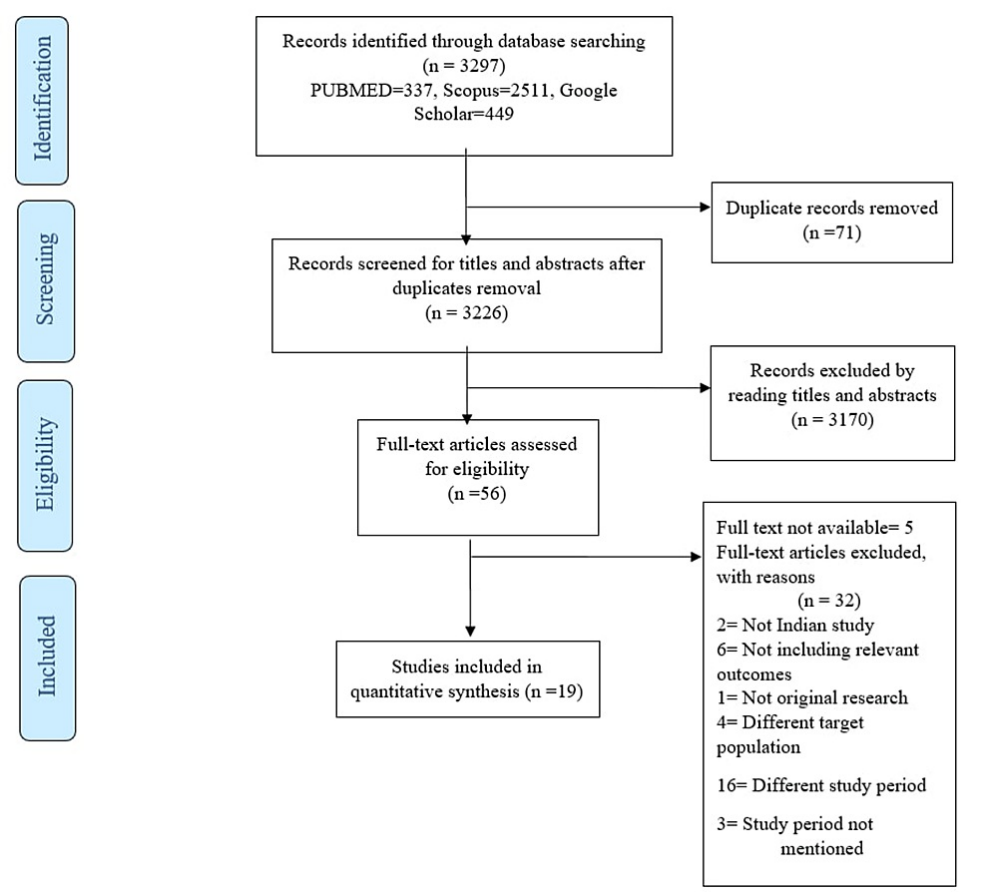
PRISMA flow diagram showing the study selection process

The initial search from PubMed, Scopus, and Google Scholar yielded 3297 studies. Only 56 pertinent papers were reviewed for eligibility after removing duplicates based on the screening of titles and abstracts. Out of 56 papers, 19 articles were included in the quantitative synthesis after various publications were excluded for multiple reasons (Figure 1).

Table 1 shows the study characteristics of the included studies comprising study population, study setting, study tool, study period, sample size and number of depressed undergraduate students along with ROB [20-38].

**Table 1:** Study characteristics of the included studies and risk of bias. NA- Not available, ROB- Risk of bias

Author	Age (years/ mean±SD)	Study population	Study setting	Study tool	Study period	Sample size	Outcome	ROB
Chakraborty A et al., 2019 [20]	17 years to 25 years	Undergraduate medical college students and interns	Sree Balaji Medical College and Hospital, Kanchipuram(dist), Tamil Nadu, India	Patient Health Questionnaire (PHQ-9)	April to June, 2019	100	90	Moderate risk
Chaudhuri A et al., 2020 [21]	18 years to 22 years	Undergraduate students	Medical College of Eastern India	Depression Anxiety Stress Scale (DASS 21)	May 2020	392	114	Low risk
Kethawath S et al., 2020 [22]	23.45± 0.7	Medical students who completed MBBS and pursue one year internship, 2019-2020	Tertiary care hospital, South India	Mental Health Literacy questionnaire	2019-2020	200	170	Moderate risk
Luthra R et al., 2020 [23]	NA	MBBS students	Private medical college of Udaipur, Rajasthan	Depression Anxiety Stress Scale (DASS 21)	September 2019	225	55	Moderate risk
Pattnaik A et al., 2020 [24]	Male:20.81±1.81Female: 20.98 ± 1.73	MBBS students from first to the fifth year	Tertiary care Government Teaching Hospital in Odisha	Patient Health Questionnaire (PHQ-9)	November 2019 to December 2019	902	902	Low risk
Prabhakar V et al., 2020 [25]	NA	MBBS students	Private medical college in north India	Depression, Anxiety and Stress Scale (DASS)	October 2019	115	55	Moderate risk
Saha R et al., 2020 [26]	NA	Undergraduate medical students	Bankura Sammilani Medical College	Beck Depression Inventory (BDI) Scale	April to June, 2019	216	63	Low risk
Vala N et al., 2020 [27]	≥17 years	1st yr MBBS	Jamnagar, Gujarat	Depression Anxiety Stress Scale (DASS 21)	-	250	39	Moderate risk
Kukreja S et al., 2021 [28]	21.28	Undergraduate medical students	Medical College situated in south Rajasthan	Beck Depression Inventory (BDI) Scale	2019	302	77	Moderate risk
Lepcha C et al., 2021 [29]	NA	1st, 2nd, 3rd, and 4th year MBBS students	Sikkim Manipal Institute of Medical Sciences, Gangtok.	hospital anxiety and depression scale (HADS)	January 2020 to January 2021	382	86	Low risk
Rana S et al., 2021 [30]	18 years to 22 years	First year MBBS students	Government Medical College, Ner Chowk, Mandi, Himachal Pradesh	Depression Anxiety Stress Scale (DASS 21)	January 2021 to March 2021	110	64	Moderate risk
Saumya J et al., 2021 [31]	NA	MBBS students	Gujarat	Patient Health Questionnaire (PHQ-9)	-	632	632	High risk
Solanki HK et al., 2021 [32]	17 years to 28 years	Undergraduate medical students	Nainital District of the Kumaon region, Uttarakhand State	Center for Epidemiologic Studies Depression Scale	June 2019 to November 2019	395	145	Moderate risk
Tomy C et al., 2021 [33]	21±1.67 years	MBBS and interns	Kerala	Depression Anxiety Stress Scale (DASS 21)	March to August 2019	500	172	Low risk
Waghmare P et al., 2021 [34]	18 years to 26 years	Undergraduate medical colleges in Maharashtra	Maharashtra	Depression Anxiety Stress Scale (DASS 21)	5th September to 12th September 2020.	435	252	Low risk
Lalithamma A et al., 2022 [35]	18 years to 20 years	1st year Medical students	Institute for physiology of the Institute for Karpaga Vinayaga Medicine, Science and Research Centre, Chinna Kolambakkam	DASS 42 questionnaire	April 2019 to May 2019	100	42	Moderate risk
Mishra J et al., 2022 [36]	18 years to 23 years	Undergraduate medical students	Kalinga Institute of Medical Sciences (KIMS), KIIT University, Bhubaneswar	Depression Anxiety Stress Scale (DASS 21)	October to November 2020	284	90	Low risk
Sasidharan A et al., 2022 [37]	18 years to 25 years	Undergraduate medical students and interns	Tertiary care Medical College and Hospital in Chennai	Depression Anxiety Stress Scale (DASS 21)	April to June 2020.	307	146	Moderate risk
Sridevi PN et al., 2022 [38]	NA	CRRI’s of a tertiary care centre, who had completed their COVID duty	Tertiary care centre, Madurai	Depression Anxiety Stress Scale (DASS 21)	October 2020 to November 2020.	97	27	Moderate risk

The Pooled Prevalence of Depression

**Figure 2:**
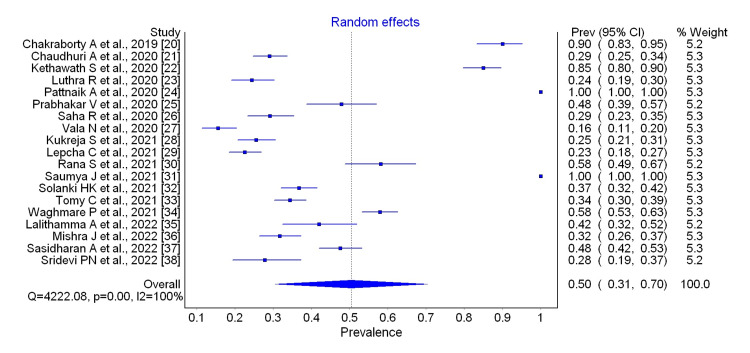
Forest plot showing the Pooled prevalence of depression among medical students

All the selected studies reported the prevalence of depression among medical students using different instruments for assessing depression. The prevalence of depression in the nineteen studies ranged from 16.0% to 100.0%. The pooled prevalence of nineteen studies, [20-38] 50.0% (95% CI: 31.0 to 70.0), was shown in Figure 2.

Minimal depression was seen in only four studies, [20,24,28,31] with a pooled prevalence of 28.0% (95% CI: 20.0 to 37.0) whereas mild depression was found in six studies, [24,27-30,34] with pooled prevalence of 14.0% (95% CI: 4.0 to 28.0). The pooled prevalence of thirteen studies that have found moderate depression was found to be 15.0% (95% CI: 11.0 to 19.0) [20,21,24,25,27-31,33-35,37]. The pooled prevalence of three studies that have found moderately severe depression was found to be 7.0% (95% CI: 3.0 to 12.0) [20,24,31]. Severe depression was seen in eleven studies [21,24,25,27-29,31,33-35,37] with a pooled prevalence of 5.0% (95% CI: 3.0 to 7.0), and extremely severe depression was found in five studies [21,25,34,35,37] with a pooled prevalence of 5.0% (95% CI: 2.0 to 11.0). Seven studies have seen the depression of undergraduate medical students among males and females. The pooled estimate of depression among the females (pooled prevalence: 38.0%, 95% CI: 20.0 to 58.0) was slightly higher when compared to males (pooled prevalence: 34.0%, 95% CI: 15.0 to 55.0). Subgroup analysis was calculated for the pooled prevalence of depression based on the instruments used for screening depression. It was found that the pooled prevalence of depression was 27.0% (95% CI: 23.0 to 31.0), 99.0% (95% CI: 96.0 to 100.0), and 37.0% (95% CI: 29.0 to 46.0) in Depression Inventory Scale, Patient Health Questionnaire, and Depression Anxiety Stress Scale respectively.

Heterogeneity and Publication Bias

**Figure 3:**
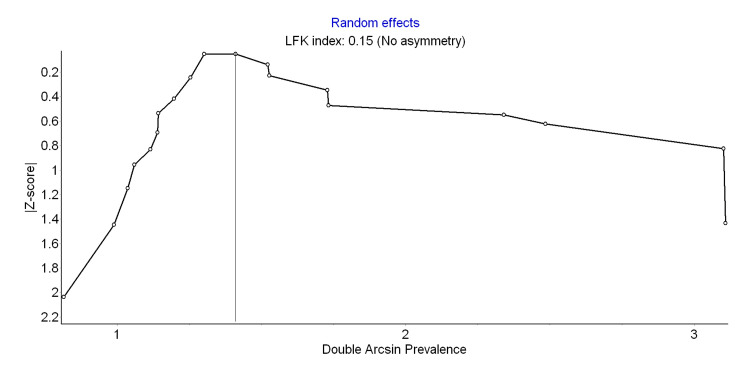
Showing Publication bias using DOI plot

The nineteen included studies were analyzed for heterogeneity and publication bias [20-38]. High heterogeneity was found in the analysis with the Q test (p <0.001) and I^2^ statistics (I^2^ = 99.6%). For publication bias, the Doi plot showed no asymmetry confirming the absence of bias (LFK index = 0.15) (Figure 3.)

Sensitivity Analysis

The effect of each study (i.e. nineteen studies) [20-41] on the pooled prevalence of depression has been analysed by excluding each study step by step using sensitivity analysis (Table 2). It showed that in eight studies, [21,23,26-29,36,38] comparatively the prime determinants of the pooled prevalence of depression among undergraduate medical students and the major source of heterogeneity come from five studies [25,30,34,35,37].

**Table 2:** Sensitivity analysis for includes studies for depression among the medical students.

Excluded study	Pooled prevalence (95% CI)	Cochran Q	I2 (95% CI)	p	
Chakraborty A et al., 2019 [20]	0.48 (0.27, 0.69)	4172.34	99.59 (99.54, 99.64)	<0.001	
Chaudhuri A et al., 2020 [21]	0.52 (0.31, 0.72)	4046.00	99.58 (99.52, 99.63)	<0.001	
Kethawath S et al., 2020 [22]	0.48 (0.28, 0.69)	4158.46	99.59 (99.54, 99.64)	<0.001	
Luthra R et al., 2020 [23]	0.52 (0.31, 0.73)	4089.99	99.58 (99.53, 99.63)	<0.001	
Pattnaik A et al., 2020 [24]	0.46 (0.30, 0.63)	2366.74	99.28 (99.16, 99.38)	<0.001	
Prabhakar V et al., 2020 [25]	0.51 (0.30, 0.71)	4214.12	99.60 (99.54, 99.64)	<0.001	
Saha R et al., 2020 [26]	0.52 (0.31, 0.72)	4128.75	99.59 (99.53, 99.64)	<0.001	
Vala N et al., 2020 [27]	0.53 (0.32, 0.73)	3975.10	99.57 (99.51, 99.62)	<0.001	
Kukreja S et al., 2021 [28]	0.52 (0.31, 0.73)	4053.56	99.58 (99.52, 99.63)	<0.001	
Lepcha C et al., 2021 [29]	0.52 (0.31, 0.73)	3962.30	99.57 (99.51, 99.62)	<0.001	
Rana S et al., 2021 [30]	0.50 (0.29, 0.71)	4221.75	99.60 (99.54, 99.64)	<0.001	
Saumya J et al., 2021 [31]	0.46 (0.28, 0.65)	2999.85	99.43 (99.35, 99.51)	<0.001	
Solanki HK et al., 2021 [32]	0.51 (0.30, 0.72)	4122.34	99.59 (99.53, 99.64)	<0.001	
Tomy C et al., 2021 [33]	0.51 (0.30, 0.72)	4066.55	99.58 (99.53, 99.63)	<0.001	
Waghmare P et al., 2021 [34]	0.50 (0.29, 0.71)	4220.50	99.60 (99.54, 99.64)	<0.001	
Lalithamma A et al., 2022 [35]	0.51 (0.30, 0.72)	4207.63	99.60 (99.54, 99.64)	<0.001	
Mishra J et al., 2022 [36]	0.52 (0.31, 0.72)	4117.93	99.59 (99.53, 99.64)	<0.001	
Sasidharan A et al., 2022 [37]	0.51 (0.30, 0.72)	4199.21	99.60 (99.54, 99.64)	<0.001	
Sridevi PN et al., 2022 [38]	0.52 (0.31, 0.72)	4177.43	99.59 (99.54, 99.64)	<0.001	

Discussion

Although it is of global public health concern, depression among medical students is still under-recognized and the associated discrimination and stigma deter most of the students from seeking help. Research done among medical students even before the pandemic were showing higher rates of depression and physical distress in comparison to the general population [3-9]. COVID pandemic may have further declined the mental health of all individuals, especially medical students.

This is one of the few systematic reviews and meta-analyses on medical trainees which has ventured into exploring the prevalence of depression amidst the COVID pandemic. It has incorporated 19 original articles published from 2019 to 2022 from medical colleges situated in different regions of India. These articles used various standard screening instruments such as Patient Health Questionnaire (PHQ-9), Depression Anxiety Stress Scale (DASS 21), Beck Depression Inventory (BDI) Scale, Centre for Epidemiologic Studies Depression Scale and Hospital Anxiety and Depression Scale (HADS) for screening depression. The pooled prevalence of depression among medical students (n=5944) was found to be 50.0% [95% CI: (31%-70%] based on a random effect model pertaining to high heterogeneity [Q test (p <0.001) and I2 = 99.6%]; the depression calculated in the various studies ranged from 16.0% to 100.0%. This pooled prevalence is higher than the pooled prevalence value of 40%, which was done before the COVID pandemic, as mentioned in a meta-analysis conducted by Dwivedi N et al. [11] In Jia Q et al. study, a meta-analysis done in 41 studies among medical students from different countries showed a high pooled prevalence of depression of 37.9% among the students. Comparatively, this meta-analysis has shown a higher prevalence among Indian medical undergraduates, slightly higher in female students [10]. This variation of the depression may reflect the trend of higher rates of depression in females in the general population [42]. The severity of depression has also been measured in various studies, though it is not uniform throughout the studies as different screening tools had different cut off for measuring depression. The pooled prevalence of depression in this review varied from 27.0% to 99.0% when stratified based on the study tools. Depression Anxiety Stress Scale was the most commonly used scale among the studies, followed by Patient Health Questionnaire and Beck Depression Inventory.

According to this meta-analysis, one out of two students was found to have some degree of depression which is a concerning number. The prevalence has increased during the COVID pandemic compared to the prevalence before the pandemic since the pandemic itself may have a negative impact on the mental health of the students [11]. Further, it was also stated that it would increase the burden of depression in South Asian populations and healthcare systems [2]. There have been multiple explanations behind depression among medical students such as the challenging medical curriculum, lack of sleep and recreational activities due to vast academic tasks given, transition from a familiar to a non-familiar situation, lack of communication skills, etc. Several strategies can be implemented at different levels like changing the grading system, setting clear learning objectives to decrease academic burden, incorporating team-based learning and group activities rather than following traditional didactic lectures, self-directed learning so that students are aware of their shortcomings and promoting professionalism and communication skills for dealing patients properly. This pandemic had led to online teaching of medical students, [43] further, the effects of social distancing and self-isolation could have made the students more vulnerable to depression and anxiety [44]. This global health concern can also be taken care of using evidence-based structured programmes for mental upliftment like mindfulness therapy, life skill training, and counselling sessions can be kept for the students [45,46].

Strengths and limitations

The strengths of this study are that it has no asymmetry in the doi plot and is one of the few meta-analyses which has investigated the pooled prevalence of depression among the undergraduate students in India. Despite the strengths, this study has a few limitations like high heterogeneity and there might be a possibility of overestimation of the prevalence of depression because pooled prevalence has been calculated based on screening instruments and in various studies which are self-reporting and does not involve any clinical diagnosis.

## Conclusions

The high prevalence of depression among medical students demands regular screening for depression along with counselling services. It shows that there is a need to raise awareness among students and other stakeholders, such as parents and medical educators, concerning symptoms and signs of depression among medical students. Further, effective strategies with health education programs and interventions may be given at individual, family, and community levels for providing mental health services to undergraduate medical students. Research with large sample sizes and multicentric longitudinal research is needed to determine the prevalence and risk factors for depression among medical students in India.

## Appendices

**Table 3:** Search strategy from databases: PubMed, Scopus, and Google Scholar.

PUBMED Search Number Query Search Details Results Time 1 Depression[MeSH Major Topic] "depressive disorder"[MeSH Major Topic] OR "depression"[MeSH Major Topic] 1,69,666 05:44:04 2 medical students[MeSH Major Topic] "students, medical"[MeSH Major Topic] 30,170 05:44:23 3 undergraduate medical students[MeSH Major Topic] ("undergraduate"[All Fields] OR "undergraduate s"[All Fields] OR "undergraduated"[All Fields] OR "undergraduates"[All Fields]) AND "students, medical"[MeSH Major Topic] 9,541 05:44:48 4 medical undergraduate[MeSH Major Topic] - Schema: all medical undergraduate[MeSH Major Topic] 0 05:45:14 5 medical undergraduate[MeSH Major Topic] medical undergraduate[MeSH Major Topic] 0 05:45:14 6 MBBS students[MeSH Major Topic] "MBBS"[All Fields] AND "students"[MeSH Major Topic] 240 05:45:27 7 India "india"[MeSH Terms] OR "india"[All Fields] OR "india s"[All Fields] OR "indias"[All Fields] 7,00,856 05:48:56 8 (((Depression[MeSH Major Topic]) AND ((medical students[MeSH Major Topic])) OR (undergraduate medical students[MeSH Major Topic])) OR (MBBS students[MeSH Major Topic])) (("depressive disorder"[MeSH Major Topic] OR "depression"[MeSH Major Topic]) AND "students, medical"[MeSH Major Topic]) OR (("undergraduate"[All Fields] OR "undergraduate s"[All Fields] OR "undergraduated"[All Fields] OR "undergraduates"[All Fields]) AND "students, medical"[MeSH Major Topic]) OR ("MBBS"[All Fields] AND "students"[MeSH Major Topic]) 9,937 05:49:56 9 ((((Depression[MeSH Major Topic]) AND ((medical students[MeSH Major Topic])) OR (undergraduate medical students[MeSH Major Topic])) OR (MBBS students[MeSH Major Topic]))) AND (India) ((("depressive disorder"[MeSH Major Topic] OR "depression"[MeSH Major Topic]) AND "students, medical"[MeSH Major Topic]) OR (("undergraduate"[All Fields] OR "undergraduate s"[All Fields] OR "undergraduated"[All Fields] OR "undergraduates"[All Fields]) AND "students, medical"[MeSH Major Topic]) OR ("MBBS"[All Fields] AND "students"[MeSH Major Topic])) AND ("india"[MeSH Terms] OR "india"[All Fields] OR "india s"[All Fields] OR "indias"[All Fields]) 337 05:56:28
SCOPUS Search number Query Results 1 ALL ( depression ) 1944890 2 ALL ( "medical students" ) 212220 3 ALL ( "mbbs students" ) 1107 4 ALL ( "undergraduate medical students" ) 10871 5 ALL ( "medical undergraduates" ) 27990 6 ALL ( "india" ) 4137884 7 ( ALL ( depression ) ) AND ( ALL ( "medical students" ) ) 26372 8 ( ALL ( depression ) ) AND ( ALL ( "mbbs students" ) ) 170170 9 ( ALL ( depression ) ) AND ( ALL ( "undergraduate medical students" ) ) 1705 10 ( ALL ( depression ) ) AND ( ALL ( "medical undergraduates" ) ) 1413 11 ( ( ALL ( depression ) ) AND ( ALL ( "medical students" ) ) ) OR ( ( ALL ( depression ) ) AND ( ALL ( "mbbs students" ) ) ) OR ( ( ALL ( depression ) ) AND ( ALL ( "undergraduate medical students" ) ) ) OR ( ( ALL ( depression ) ) AND ( ALL ( "medical undergraduates" ) ) ) 26596 12 ( ( ( ALL ( depression ) ) AND ( ALL ( "medical students" ) ) ) OR ( ( ALL ( depression ) ) AND ( ALL ( "mbbs students" ) ) ) OR ( ( ALL ( depression ) ) AND ( ALL ( "undergraduate medical students" ) ) ) OR ( ( ALL ( depression ) ) AND ( ALL ( "medical undergraduates" ) ) ) ) AND ( ALL ( "india" ) ) 3306 13 ( ( ( ALL ( depression ) ) AND ( ALL ( "medical students" ) ) ) OR ( ( ALL ( depression ) ) AND ( ALL ( "mbbs students" ) ) ) OR ( ( ALL ( depression ) ) AND ( ALL ( "undergraduate medical students" ) ) ) OR ( ( ALL ( depression ) ) AND ( ALL ( "medical undergraduates" ) ) ) ) AND ( ALL ( "india" ) ) AND ( LIMIT-TO ( DOCTYPE , "ar" ) ) 2511
Google scholar allintitle: medical students depression 449
